# The impact of a novel medication scanner on administration errors in the hospital setting: a before and after feasibility study

**DOI:** 10.1186/s12911-022-01828-3

**Published:** 2022-03-29

**Authors:** Clare L. Tolley, Neil W. Watson, Andrew Heed, Jochen Einbeck, Suzanne Medows, Linda Wood, Layla Campbell, Sarah P. Slight

**Affiliations:** 1 School of Pharmacy, Newcastle University, Newcastle Upon Tyne, UK; 2 The Newcastle Upon Tyne Hospitals NHS Foundation Trust, Newcastle, UK; 3 Department of Mathematical Sciences, Durham University, Durham, UK; 4 Durham Research Methods Centre, Durham University, Durham, UK; 5 The Centre for Patient Safety Research and Practice, Division of General Internal Medicine and Primary Care, Brigham and Women’s Hospital, Boston, MA USA

**Keywords:** Patient safety, Medication errors, Medication administration, Health care systems

## Abstract

**Objective:**

The medication administration process is complex and consequently prone to errors. Closed Loop Medication Administration solutions aim to improve patient safety. We assessed the impact of a novel medication scanning device (MedEye) on the rate of medication administration errors in a large UK Hospital.

**Methods:**

We performed a feasibility before and after study on one ward at a tertiary-care teaching hospital that used a commercial electronic prescribing and medication administration system. We conducted direct observations of nursing drug administration rounds before and after the MedEye implementation. We calculated the rate and type (‘timing’, ‘omission’ or ‘other’ error) of medication administration errors (MAEs) before and after the MedEye implementation.

**Results:**

We observed a total of 1069 administrations before and 432 after the MedEye intervention was implemented. Data suggested that MedEye could support a reduction in MAEs. After adjusting for heterogeneity, we detected a decreasing effect of MedEye on overall errors (*p* = 0.0753). Non-timing errors (‘omission’ and ‘other’ errors) reduced from 51 (4.77%) to 11 (2.55%), a reduction of 46.5%, which had borderline significance at the 5% level, although this was lost after adjusting for confounders.

**Conclusions:**

This pilot study detected a decreasing effect of MedEye on overall errors and a reduction in non-timing error rates that was clinically important as such errors are more likely to be associated with harm. Further research is needed to investigate the impact on a larger sample of medications.

**Supplementary Information:**

The online version contains supplementary material available at 10.1186/s12911-022-01828-3.

## Introduction

The medication administration process is complex and influenced by interruptions, multi-tasking and responding to patient’s care needs [1, 2]. It is consequently prone to errors, with over half (54.4%) of the 237 million medication errors estimated to have occurred in England each year taking place at the administration stage; 7.6% of these were associated with moderate or severe harm [3]. A large systematic literature review reported an overall Medication Administration Error (MAE) rate of 19.6% in the hospital and long-term care settings, with just over half of these related to timing [4]. Keers et al. identified how slips and lapses commonly occurred at the medication administration stage and how these incidents were often associated with the working environment, busy working conditions and distractions [5].

The implementation of Health information Technology (HIT) as part of a Closed Loop Medication Administration (CLMA) solution aims to reduce medication administration errors and prevent patient harm. Interventions such as Barcode Medication Administration (BCMA), electronic prescribing, and scanning of patient barcodes, have all been associated with reduced errors and harm, and improved efficiency [1, 6, 7]. Systems that can perform checks on solid oral dosage forms (rather than relying on a barcode) are also available. This may avoid issues identifying medicines where the barcode is not available, e.g., if the original packaging has been disposed of, or if a blister pack of medicines has been placed inside the wrong outer packaging. MedEye is a novel medication scanner, which can perform checks on solid dosage forms using image detection from the scanner camera, against a reference image database to prevent medication administration errors occurring at the bedside, however there is a lack of evidence about the effectiveness of such tools. We therefore conducted a feasibility study to investigate the impact of MedEye on the rate of medication administration errors in a hospital setting and whether further investigation would be promising.

## Materials and methods

### Setting

This study was conducted on a 30-bed respiratory medical ward at a tertiary-care teaching hospital Trust. Typically, one nurse would care for one bay of 6 patients with support from a health care assistant. Drug administration rounds usually occurred on this ward four times a day at approximately 7.45 am, 12 pm, 6 pm and 10 pm. One or two ward-based nurses, would administer medicines for half of the ward and a separate set of nurses would administer medicines for the other half. These nurses administered medications alongside other roles such as: performing clinical tests and assessments, personal care and patient planning. All medicines that corresponded to the drug administration round times would be administered, including PRNs, injectables and controlled drugs etc.. All medications were ordered electronically and administration documented by nurses on a commercial electronic prescribing and medication administration (ePMA) system. The ePMA system had been in place for over 10 years and included some clinical decision support at the administration stage (e.g., an alert generated if a nurse attempted to administer paracetamol within 4-h of the last dose). Nurses administered medications from boxes that were either stored in patient bedside lockers and/or in a central ward medication cupboard. The patient bedside lockers included medications that: the patient had brought in from home, were supplied by the hospital pharmacy and/or supplied from the ward. It could not be guaranteed that all items within the lockers had been verified by a member of the pharmacy team. The central ward medication cupboard was a non-electronic cupboard that contained medicines listed in the ward’s medication formulary. The cupboard contained shelves of medicines that were typically arranged alphabetically and stocked by pharmacy. This study was approved by the North East—Tyne & Wear South Research Ethics Committee (17/NE/0342).

### Intervention

**Fig. 1:**
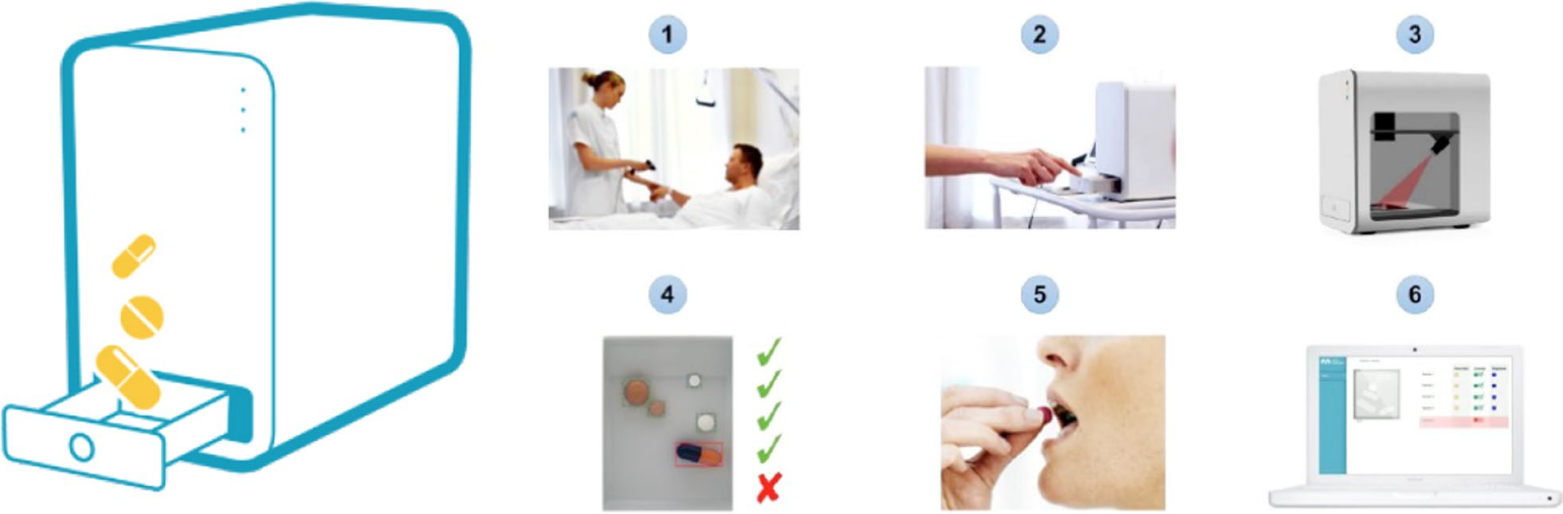
MedEye Medication Pill Scanner ( *Source* MedEye reproduced with permissions)

**Fig. 2:**
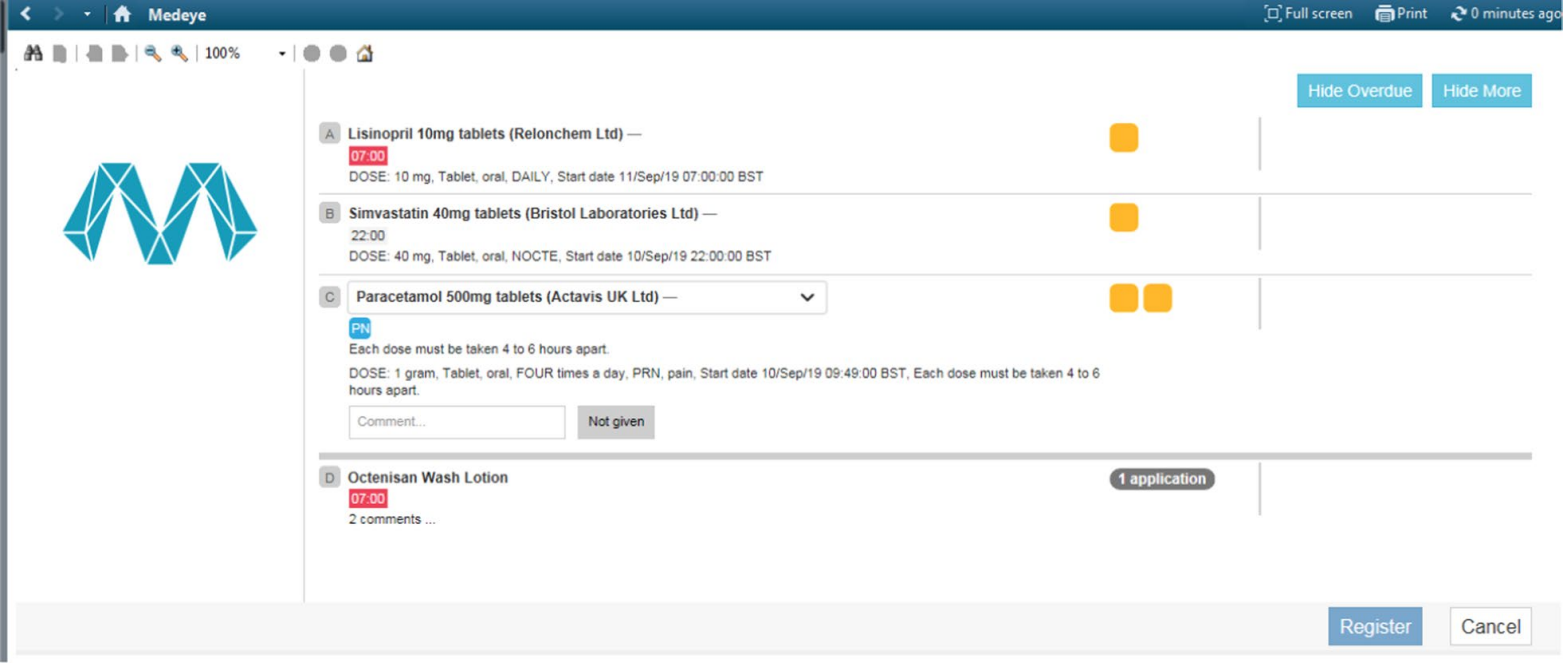
Example of standard MedEye nursing drug administration drug chart view ( *Source* MedEye: reproduced with permissions)

MedEye is a medication scanner, used at the bedside. Prior to administering medications, nurses were required to confirm the patient’s identity by asking the patient for their name and date of birth, which was cross-referenced against their health record. Nurses would open the MedEye window from within the hospital ePMA system (MedEye was integrated into the hospital’s main ePMA system so no additional log-in step was needed), and see what medication the patient was prescribed/due and then place that medicine(s) (solid oral dosage form) on the MedEye scanning tray (see Fig. 1). The MedEye scanner then scanned the medication to identify the type and quantity of medicines in the tray, and cross referenced this information with the patient’s EHR. The nurse would be notified if the medicine presented was not prescribed/due. If the dose was correct, the MedEye system would acknowledge this and the nurse would click a button to register the medication as administered. Any medicine(s) that was ordered using free-text, IVs and/or liquids could not be verified using the MedEye scanner and were instead recorded manually by the nurse in the MedEye user interface or separately on the hospital ePMA system. The MedEye user-interface presented nurses with a condensed list of medications with doses to be given at that time. Therefore, nurses administering medications at 7.30am would only see doses due to be given as part of a patient’s morning regimen (anything prescribed between 5.30am – 9am; these default settings were configurable) thus potentially reducing the cognitive screen burden (see Fig. 2).

### Study design

**Fig. 3:**
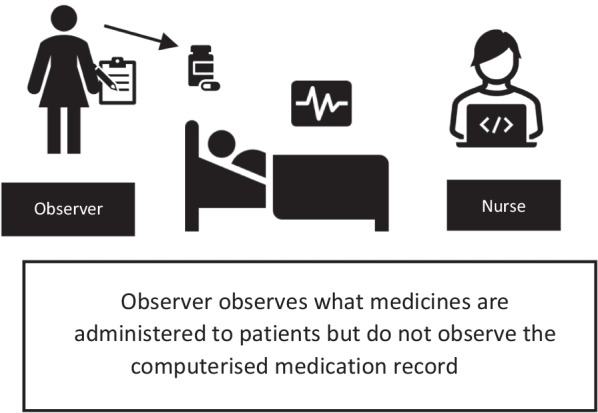
Diagram of observation

We conducted a before-and-after feasibility study to compare the number and type of medication administration errors 4–6 months before and 1 week after the intervention was implemented. Nurses received hands-on training prior to the roll-out of MedEye, which consisted of: (a) attending an information session concerning the system’s functionality, (b) orientation of the MedEye user-interface, and (c) practical training and experience of using the system on test patients. The nurses were supervised and supported by a trainer (either a super-user or representative from the MedEye deployment team) on a minimum of three drug rounds and/or until the nurse was confident enough to use the system. Observer training consisted of attending a meeting that gave an overview of the project, reading the study protocol and data collection forms, and attending at least three pre-study pilot observation sessions, accompanied by a member of the research team. We used a previously validated method of data collection outlined by Barker et al. [11], During data collection, one observer (registered nurse or pharmacist) shadowed the nurse responsible for drug administrations on each of these ward rounds and made a note of what they observed being *administered* to each patient (see Additional file 1 for a copy of the data collection tool used). They were blinded at the time to what was *prescribed* for each patient, so as to avoid being influenced by the electronic order. In other words, the observer could see what was administered to the patient (e.g., 1 × bisoprolol 2.5 mg tablet) given either with or without the MedEye scanning device, but did not have access or could not see the patient’s electronic drug chart to check what was prescribed (see Fig. 3), thus serving to detect the actual error rate. After completing each ward round, the observer reviewed all prescribed orders in the eMAR and compare what was *prescribed* with what was *administered*. We included observations of all solid-oral dosage forms that could be given by nurses, doses that were ‘self-administered’ by patients were excluded.

We used a previously established definition of a MAE to be “*any dose of medication that deviated from the patient’s current medication orders”* [8]*.* Errors were grouped into three main categories: ‘timing’ errors (administrations that were early or late by between 1–2 h or over 2 h), ‘omission’ errors and ‘other’ errors, the latter including: wrong patient, wrong administration equipment used, wrong dose, wrong route, wrong medication, documentation error, administration without order, failure to recognise drug-drug interaction, patient had a documented allergy to medication, directions/ monitoring error. The denominator was the number of opportunities for error (OE) defined as all solid oral doses administered plus any doses omitted [8]. Each error recorded by the observer was reviewed independently by two trained observers (nurse and/or pharmacist) who confirmed the presence of an error after the point of administration, classified the error type according to a pre-defined list, and assessed the potential of that error to lead to patient harm. Disagreements were resolved through discussion and involvement of a further member of the study team. This ensured consistent classification of the error, error type and potential to lead to harm.

Pre-MedEye data collection occurred between August and November 2019, post-MedEye data collection occurred between February and March 2020, but ended early due to the COVID-19 pandemic. We had originally aimed to observe around 1000 administrations pre-MedEye and 1000 administrations post-MedEye on the study ward, and had originally planned to collect data at least 4 weeks after MedEye was implemented. All research was performed in accordance with relevant guidelines/regulations, and informed consent was obtained from nurse participants who were observed. Research involving human research participants has been performed in accordance with the Declaration of Helsinki. The datasets used and/or analysed during the current study are available from the corresponding author on reasonable request.

### Analysis

Tests on equality of rates were carried out using exact unconditional tests for 2 × 2 contingency tables [9] which are the more adequate choice than traditional chi-squared tests in the presence of small counts. 95% confidence intervals were obtained alongside the tests through this methodology. For the subcategories of timing errors, error rates were considered both in relation to overall opportunities for error, as well as in relation to overall timing errors. The same principle was followed for omission errors due to lack of ward stock.

To adjust for heterogeneity and correlation effects due to nurses, patients, or observers (i.e., clustering effects in the data arising from within-observer-correlations), we fitted a binomial (logit) mixed effect model for each error type. Specifically, the occurrence or non-occurrence of the respective error served as a dichotomous response variable (with one observation corresponding to one OE). A two-factor fixed effect variable (`period’, with values `before’ and `after’) was included to capture the effect of the implementation of the Medeye System, along with another fixed effect variable for nurse time on duty at the time of medication administration (in hours). Random effects were included for each nurse, each patient, and also for the first and/or second observer, if a prior Analysis of Deviance indicated any observer effects and was omitted if a prior analysis of deviance indicated the absence of such effects. The random effect was taken to be Gaussian, and the models were fitted using R function glmmTMB [10].

## Results

Trained observers directly observed 35 drug rounds (morning, lunchtime and evenings on weekdays) [11]. We observed 1,069 administrations before and 432 after MedEye intervention was implemented (see Table 1). These administrations were given by a total of 19 different nurses, and the majority were staff nurses in the age ranges of between 26–35 (47%, n = 9) and 46–55 (26%, n = 5) years old, just over a quarter of nurses (26%, n = 5) had been qualified for 5 years or less, 32% (n = 6) had been qualified for between 6–15 years and 42% (n = 8) had been qualified for between 16 and 40 years.

**Table 1:** Rates of medication administration errors

Type of error	Pre-intervention number of errors (% of OE)	Post-intervention number of errors (% of OE)	95% CI*	*p *value*	95% CI (adjusted for heterogeneity) for the coefficient in the fitted logit model corresponding to the `period' indicator. For the difference in error rate pre- and post-intervention†	*p *value (adjusted for heterogeneity) †
Total errors	739 (69.1)	302 (69.9)	− 4.6 to 5.9%	0.773	− 2.2 to 0.1	0.0753
Non-Timing errors	51 (4.77)	11 (2.55)	− 4.2% to 0.1%	0.050	− 2.0 to 0.2	0.115
Omission errors	17 (1.6)	4 (0.9)	− 1.9% to 0.9%	0.326	− 2.3 to 1.3	0.579
Omission errors due to lack of ward stock	9 (0.8)	3 (0.7)	− 1.1% to 1.3%	1.0	− 3.4 to 3.3	0.976
(% of total number of omission errors in that period)	− 52.9	− 75	− 30.6% to 55.6%	0.486		
Other administration errors	34 (3.2)	7 (1.6)	− 3.2% to 0.4	0.096	− 2.6 to 0.8	0.295
Wrong Dose	1 (0.09)	0 (0.0)	− 0.6% to 0.8%	1.0	Result could not be generated	-
Documentation Error	28 (2.6)	7 (1.6)	− 2.5% to 0.9%	0.263	− 3704.1 to 3728.2	0.9949***
Wrong Form	5 (0.5)	0 (0.0)	− 1.1% to 0.5%	0.183	− 1714.3 to 1694.2	0.9908***
Timing errors	688 (64.4)	291 (67.4)	− 2.6% to 8.3%	0.282	− 1.6 to 0.5	0.318^¢^
Early 1–2 h% of timing errors	25 (2.3)	34 (7.9)	3.1% to 8.6%	< 0.00001	3.1 to 15.7	0.00341
	− 3.6	− 11.7	4.4% to 12.6%	< 0.00001		
Early > 2 h% of timing errors	3 (0.28)	0 (0.0000)	− 0.9% to 0.7%	0.286	− 247,877.8 to 247,831.9	0.9999
	(0.44)	(0.0000)	1.3% to 1.0%	0.271		
Late 1–2 h% of timing errors	568 (53.1)	229 (53.0)	− 5.8% to 5.5%	0.9711	− 1.3 to 2.7	0.493^‡^
	− 82.6	− 78.7	− 9.7% to 1.5%	0.159		
Late > 2 h% of timing errors	92 (8.6)	28 (6.5)	− 4.9% to 1.0%	0.174	− 8.0 to − 0.7	0.0188
	− 13.4	− 9.6	− 7.9% to 0.9%	0.103		

OE- opportunity for error

*p value reported using the Z-pooled Exact Test (exact unconditional tests for 2 × 2 contingency tables)

^†^*p *value adjusted for heterogeneity including possible correlation effects within nurses, patients, and observers

^¢ ^We did not identify a significant impact of MedEye on the overall rate of timing errors but did note a significant decrease with nurse time on duty. It is possible this was due to the busier morning drug rounds, which increased the likelihood of nurses making a mistake

^‡^We also noted a strongly significant decreasing effect of nurse time on duty on late 1–2 h errors. This was possibly associated with calmer and quieter drug rounds that occurred later in the day

***Fitted models do not include Nurse time on duty as models do not fit otherwise

°There were no reports of the following ‘*other—error subtypes’:* wrong patient, wrong administration equipment used, wrong medication error, administration without order, route error, failure to recognise drug-drug interaction, patient had a documented allergy to medication, directions/ monitoring error

An overview of MAE rates is shown in Table 2. The percentage of MAEs pre-MedEye (69.1%) and post-MedEye (69.9%) remained almost the same. However, after adjusting for heterogeneity, we did detect some weak evidence for the decreasing effect of MedEye on overall errors (*p* = 0.0753).

**Table 2:** Examples of medication administration errors

Error type	Pre-intervention	Post-intervention
Timing error	Sertraline prescribed for 7am but not given until 8.30am	Rifaximin prescribed for administration at 7am but not given until 9.02am
Omission error	Aspirin prescribed but mistakenly omitted	Cinacalcet not in stock therefore knowingly omitted
Documentation error	Patient refused memantine but recorded as administered on the system	Gabapentin administered to a patient but nurse did not register this on the system
Wrong dose	Nurse was about to give 40 mg of furosemide but 20 mg prescribed (observer intervened)*	–
Wrong form	Modified release metformin prescribed but standard release given	–

*Although observers were blinded to the patient’s medication chart there were instances were observers visited patients on multiple occasions and therefore were aware of some of their medicines and so may have been able to intervene if they encountered an issue

### Non-timing errors (omission and other errors)

Non-timing errors reduced from 51 (4.77%) to 11 (2.55%) of the Opportunities for Error (OE), which was statistically significant (*p* = 0.05) based on the z-test analysis. However, this was not found to be significant after adjusting for nurse, patient and observer.

We recorded a non-significant fall in omission errors from 17 (1.6%) pre-MedEye to 4 (0.9%) post-MedEye (z test: *p* = 0.326, adjusted value: 0.579). Of these omission errors, a large proportion were due to a lack of ward stock, accounting for 9 (52.9%) and 3 (75%) of the total number of omission errors pre- and post-MedEye, respectively; this difference was not statistically significant (*p* = 0.486). We also saw a non-significant reduction in ‘other’ error types (e.g., dose and documentation errors) following the implementation of MedEye from 34 (3.2%) to 7 (1.62%), (z-test: *p* = 0.096, 95% CI − 3.2 to 0.4% and adjusted values: *p* = 0.295). In particular, there were non-significant reductions in documentation errors from 28 (2.6%) to 7 (1.6%), and wrong formulation errors from 5 (0.5%) to 0 (0%), pre- and post-MedEye, respectively. There were no reports of wrong patient, wrong administration equipment used, wrong medication error, administration without order, route error, failure to recognise drug-drug interaction, patient had a documented allergy to medication, directions/monitoring error. It is worth noting that an observer witnessed a nurse dispense the wrong medication (prednisolone) instead of the intended medication (furosemide) in the post-MedEye period. After receiving a notification from MedEye that an unexpected medication had been dispensed, the nurse corrected the dose thus preventing the error from reaching the patient. We also identified one instance where the nurse correctly dispensed a prescribed medication (amlodipine) but this was mistakenly identified by the MedEye scanner as another medication that was prescribed (metoclopramide). In this case, the medication metoclopramide was wrongly recorded as administered on the system instead of amlodipine, resulting in a documentation error.

### Timing errors

Timing errors were found to be the most common error type in both periods accounting for 688 (64.4%) of the OE pre-MedEye and 291 (67.4%) of the OE post-MedEye. There was no change in this overall rate of timing errors following the intervention (z-test: *p* = 0.282, 95% CI − 2.6% to 8.3%) (adjusted value: *p* = 0.318). The majority of timing errors were associated with doses that were given between 1–2 h after the prescribed time (e.g., bisoprolol prescribed for 7am as per the default prescription times but not administered until 8.27am, due to the ward workflow). However, we did find an increase in the rate of doses administered 1–2 h earlier than the prescribed time from 25 (2.3%) pre-MedEye to 34 (7.9%) post-MedEye, *p* =  < 0.00001, 95% CI 3.1% to 8.6%, which remained significant after adjusting for confounders. Hospital policy states that medications should be given within 2 h before or after the prescribed time (i.e., if gabapentin was prescribed for 7am, the dose was considered overdue if it was given after 9am unless there was a clinical justification for the delay). Reassuringly, we found relatively few doses (between 0—8.6% of all administrations) that were given outside of this 2-h drug administration window. In total, 3 (0.3%) of all administrations were given 2-h earlier pre-MedEye and 0 (0%) post-MedEye, while 92 (8.6%) and 28 (6.5%) of all administrations were given 2-h later pre and post-MedEye, respectively. This decrease was statistically significant after adjusting for heterogeneities (*p* = 0.0188). Examples of the types of MAEs can be found in Table 2.

## Discussion

We conducted the first feasibility evaluation of MedEye to assess its impact on the rate of medication administration errors experienced with solid oral dosage forms on a 30-bed respiratory ward, to inform future investigation. Our study suggested that the methodology was feasible and also found a slightly decreasing effect on the overall MAE rate after the MedEye implementation. We did find a borderline significant reduction in non-timing errors (combination of ‘omission’ + ‘other’ errors); however, after adjusting for confounders, significance was lost suggesting that the results may have been coincidental. The overall rate of timing errors was unaffected. Additional data collection was not feasible within the project timeline, due to limits of research activity during the COVID 19 pandemic, however the data does suggest that further investigation would be promising. See Table 3 for a summary table of what was already known on the topic and what this study added to our knowledge.

**Table 3:** Summary Table

What was already known on the topic	What this study added to our knowledge
Barcode medication administration technology has been shown to improve patient safety when used properly	Direct observations can be used to evaluate the effectiveness of closed loop medication administration (CLMA) systems
There may be instances where barcode scanning of medications is not possible e.g., barcode label missing, unit dose dispensing, and alternative verification approaches are of interest	Systems that scan the solid oral dosage units may contribute to reduced medication administration errors as part of a CLMA process
	This study is the first evaluation of MedEye in a UK hospital setting

A reduction in the overall MAE, and in particular non-timing errors, is notable because incidents within this category e.g., dose errors, are more likely to be associated with harm compared to timing errors [12]. Timing errors were also heavily influenced by hospital policy, (e.g., the default drug administration times specified in the system), and nursing workflow (e.g., the time nurses finished handover or other tasks to begin administering medications). Consequently, these were less likely to be affected by the MedEye system. Changes to the ePMA system are needed to more accurately reflect the nursing workflow, for example modifying scheduled drug administration times to correspond with the typical times that nurses administered medications in different clinical areas. The scale of the reduction in non-timing (omission + other) errors was in-line with other studies that have implemented systems as part of a CLMA process [8, 12].The pre- and post- non-timing error rate was 4.77% and 2.55% respectively, which was lower than the non-IV error rate of 10.1% and 4.5% reported by Dean-Franklin et al., before and after implementing a closed‐loop electronic prescribing, automated dispensing, barcode patient identification and EMAR system in one surgical ward in a London teaching hospital [8, 13]. Similarly, Poon et al., reported a non-timing error rate of 11.5% on wards that did not use BCMA compared to a non-timing MAE rate of 6.8% on wards that did [12]. Our baseline non-timing error rates were likely to be lower than those reported in these previous two studies because nurses at our site already used a well-established ePMA system for over 10 years and were therefore familiar with using technology. In addition, we did not include errors associated with intravenous or liquid-dosage forms as these were beyond the study scope. However, these types of errors may be more prone to calculation errors in the dispensing and administration stages and are more likely to cause patient harm [14, 15]. Further work is needed to explore the impact of using the MedEye scanner alongside other verification tools (e.g., barcoding), other visual verification approaches (e.g., digital second verification) and smart pump technology for injectables and liquid dosage forms [16–18].

Other systems such as BCMA and automated dispensing cabinets have been associated with reductions in medication administration errors [19, 20]. However, studies have also reported error-prone practices associated with this technology. For example, a study conducted across four hospitals in the Netherlands revealed how procedural workarounds, such as not scanning medications, were commonly encountered [21]. This was related to a higher patient to nurse ratio, with nurses employing workarounds more often when they were busier [21]. It is important that technology is compatible with the user’s existing workflow and is not burdensome, to avoid adding additional and unacceptable steps to their routine. Factors related to the MedEye system’s design may have contributed to the reduction in ‘omission’ and ‘other’ error types, which may have contributed to the borderline significant reduction in the overall MAE rate that we found. In particular, the user-interface presented a simplified list of the patient’s medications, preventing nurses from overlooking doses that were due and accidently omitting prescriptions from a long list that they needed to scroll through. The design of systems is important and features such as lengthy menu lists can contribute to errors [22, 23]. The MedEye user interface also allowed the nurse to register the administration of groups of medications by clicking one button when compared to the previous ePMA system interface, which required nurses to register each individual medication sequentially; in other words, if a patient was on 10 medications, this would require 10 separate ‘sign off’ tasks. Poor system usability, which requires users to go through multiple steps to complete a task has been reported in the literature to contribute to medication errors and clinician burnout [24]. We observed one example where MedEye mis-identified one medication as another and this in turn resulted in a documentation error. Other studies have also found that technology may contribute to medication errors and unintended consequences, thus post-implementation evaluation is important to understand the causes and identify ways to prevent such errors from re-occurring [23, 25, 26].

This feasibility study was the first evaluation of MedEye on the rate of MAEs using direct observational methods. However, there are some limitations of our work. Our study took place on one UK hospital ward, thus potentially limiting the generalisability of our findings to other settings. It is also possible that nurses altered their behaviour when they were being observed due to the Hawthorne effect; however, the researchers did take steps to prevent this and the extended periods of observations were likely to have reduced this impact [27]. The reduced sample of post-implementation data collected as a result of the COVID 19 pandemic also affected our ability to derive significant findings. Due to the sudden nature in which restrictions were placed due to the COVID 19 pandemic, we were not able to adjust the data collection methods to increase the quantity of post-intervention data. Further research should aim to expand the number of medication administrations observed post-MedEye. Further work should also investigate the impact of MedEye and related technology on intravenous and oral liquid associated medication errors and on workarounds. Since the study was conducted MedEye functionality has increased to include checks for unmarked inhalers and barcoded medications (further information can be accessed via the MedEye company website: https://medeye.com/). In addition, further research is needed to evaluate data retrieved from MedEye on the discrepancies detected. Due to time pressures, we conducted the first round of post-MedEye data collection 1 week after they had been trained to use the system. Although a longer embedding phase would have been preferred, this still gave us the opportunity to evaluate the early impact of the system on medication administration errors. This work will be considered alongside research performed to consider the impact of the system on nursing time and the barriers and facilitators to the MedEye implementation process. Additional work is also needed to explore the impact of the system on nurse satisfaction as this is an important factor related to the adoption and long-term use of new systems in practice.

## Conclusions

This before and after feasibility study is the first evaluation of a novel medication scanning device, MedEye on the rate of MAEs in one of the largest NHS trusts in England. We found a reduction in non-timing error rates that was clinically important as such errors are more likely to be associated with harm. Further research is needed to investigate the impact of MedEye on a larger sample of medications.

## Supplementary Information

**Additional file 1.** Administration Error Classification Form.

## Data Availability

The datasets used and/or analysed during the current study available from the corresponding author on reasonable request.
