# Supplementary material for: The impact of a novel medication scanner on administration errors in the hospital setting: a before and after feasibility study
Source: BMC Med Inform Decis Mak. 2022 Mar 29;22:86. doi: 10.1186/s12911-022-01828-3 (PMC8962937; doi:10.1186/s12911-022-01828-3)
Supplement: Supplementary file 1 — Additional file 1. Administration Error Classification Form. [file 12911_2022_1828_MOESM1_ESM.doc]

| **PART 1: Error Classification** | | |
| --- | --- | --- |
| 1. **Date error identified** | |  |
| 1. **Ward ID code (e.g., HOS/W2)** | |  |
| 1. **Administration ID Code (Hospital/Ward/ Patient Number/Incident letter) (e.g., HOS/W2/001/a)** | |  |
| 1. **Initials of Observer:** | |  |
| 1. **Participant Nurse ID:** | |  |
| 1. **Initials of Reviewer:** | |  |
| 1. **Number of medications ordered for that patient in the last 24 hour period** | |  |
| 1. **Number of other medications given at the time of error** | |  |
| 1. **Details of Error** | | ……………………………………………………………………  ……………………………………………………………………  ……………………………………………………………………  …………………………………………………………………… |
| 1. **Name of Medication** | |  |
| 1. **Classification of Medication** | | **Tick ONE option below** |
| | **[ ]** | 1. ACE Inhibitor | **[ ]** | 1. Antihypertensive | **[ ]** | 1. Digoxin | **[ ]** | 1. Peptic Ulcer | | --- | --- | --- | --- | --- | --- | --- | --- | | **[ ]** | 1. Analgesic (narcotic) | **[ ]** | 1. Antipsychotic | **[ ]** | 1. Diuretics | **[ ]** | 1. Sedative/ Anxiolytic | | **[ ]** | 1. Analgesic (non-narcotic) | **[ ]** | 1. Antiepileptic | **[ ]** | 1. Electrolyte concentrates | **[ ]** | 1. Steroid | | **[ ]** | 1. Anti-Parkinson | **[ ]** | 1. Anti-cancer | **[ ]** | 1. Hormonal replacement therapy | **[ ]** | 1. Synthroid | | **[ ]** | 1. Antiarrhythmic | **[ ]** | 1. Beta-blocker | **[ ]** | 1. Mucolytic | **[ ]** | 1. Theophylline | |  | 1. Antibiotic | **[ ]** | 1. Calcium channel blocker | **[ ]** | 1. Muscle relaxants | **[ ]** | 1. Vitamins | |  | 1. Anticoagulant |  | 1. Bronchodilators |  | 1. Nitrates | **[ ]** | 1. Other________________________ | | **[ ]** | 1. Antidepressant | **[ ]** | 1. Cholesterol lowering medication | **[ ]** | 1. NSAID |  | BLANK | | **[ ]** | 1. Antihistamine | **[ ]** | 1. Anti-diabetes | **[ ]** | 1. Oral contraceptive |  | BLANK | | | |
| 1. **Medication Status** | New [ ] Chronic [ ] | |
| 1. **Was the error an omission error** | **Yes [ ]**  **No [ ]** | |
| 1. **Was the error a timing error?** | **Yes [ ]**  **No [ ]** | |
| 1. **If yes to question 12, was the timing error a?** | **(a) [ ] Early administration error**  [ ] 1 hour early  [ ] 1-2 hours early  [ ] >2 hours early  **(b) [ ] Late administration error**  [ ] 1 hour late  [ ] 1-2 hours late  [ ] >2 hours late | |
| 1. **If NOT an omission or timing error what was the category of administration error. (Choose ONE of the following)** | | **a) [ ] Wrong Patient** | **f) [ ] Wrong Medication Error** | | --- | --- | | **b) [ ] Wrong administration equipment used *(e.g., a spoon instead of syringe)*** | **g) [ ] Administration without order *(e.g., drug given but was not prescribed)*** | | **c) [ ] Administration documentation error *(e.g., documented as given but was not)*** | **h) [ ] Route Error** | | **d) [ ] Dose Error**  [ ] Incorrect dosage form  [ ] Overdose  [ ] Underdose  [ ] Extra dose  [ ] Incomplete dose  [ ] Other  …………………………………………….. | **i) [ ] Patient had a documented allergy to medication prescribed** | | **e) [ ] Failure to recognise drug-drug interaction** | **j) [ ] Directions/ Monitoring Error** | | |
